# High-affinity CD8 variants enhance the sensitivity of pMHCI antigen recognition *via* low-affinity TCRs

**DOI:** 10.1016/j.jbc.2023.104981

**Published:** 2023-06-28

**Authors:** Lea Knezevic, Tassilo L.A. Wachsmann, Ore Francis, Tamsin Dockree, John S. Bridgeman, Anne Wouters, Ben de Wet, David K. Cole, Mathew Clement, James E. McLaren, Emma Gostick, Kristin Ladell, Sian Llewellyn-Lacey, David A. Price, Hugo A. van den Berg, Zsuzsanna Tabi, Richard B. Sessions, Mirjam H.M. Heemskerk, Linda Wooldridge

**Affiliations:** 1 Faculty of Health Sciences, University of Bristol, Bristol, UK; 2 Department of Haematology, Leiden University Medical Center, Leiden, The Netherlands; 3 Division of Infection and Immunity, Cardiff University School of Medicine, University Hospital of Wales, Cardiff, UK; 4 Instil Bio Inc, Dallas, Texas, USA; 5 Immunocore, Abingdon, UK; 6 Systems Immunity Research Institute, Cardiff University School of Medicine, University Hospital of Wales, Cardiff, UK; 7 Warwick Mathematics Institute, University of Warwick, Coventry, UK; 8 Faculty of Life Sciences, University of Bristol, Bristol, UK

**Keywords:** CD8, human leukocyte antigen (HLA), immunotherapy, major histocompatibility complex (MHC), T cell receptor (TCR)

## Abstract

CD8^+^ T cell-mediated recognition of peptide-major histocompatibility complex class I (pMHCI) molecules involves cooperative binding of the T cell receptor (TCR), which confers antigen specificity, and the CD8 coreceptor, which stabilizes the TCR/pMHCI complex. Earlier work has shown that the sensitivity of antigen recognition can be regulated *in vitro* by altering the strength of the pMHCI/CD8 interaction. Here, we characterized two CD8 variants with moderately enhanced affinities for pMHCI, aiming to boost antigen sensitivity without inducing non-specific activation. Expression of these CD8 variants in model systems preferentially enhanced pMHCI antigen recognition in the context of low-affinity TCRs. A similar effect was observed using primary CD4^+^ T cells transduced with cancer-targeting TCRs. The introduction of high-affinity CD8 variants also enhanced the functional sensitivity of primary CD8^+^ T cells expressing cancer-targeting TCRs, but comparable results were obtained using exogenous wild-type CD8. Specificity was retained in every case, with no evidence of reactivity in the absence of cognate antigen. Collectively, these findings highlight a generically applicable mechanism to enhance the sensitivity of low-affinity pMHCI antigen recognition, which could augment the therapeutic efficacy of clinically relevant TCRs.

CD8^+^ T cells recognize peptide fragments bound to surface-expressed major histocompatibility complex class I (MHCI) molecules *via* somatically rearranged T cell receptors (TCRs). This process of antigen recognition triggers an array of effector functions, including direct cytotoxicity, which act synergistically to eliminate infected or transformed cells from the body. Autoreactive or cancer-targeting TCRs typically display low to moderate affinities for cognate pMHCI, whereas pathogen-specific TCRs typically display high affinities for cognate pMHCI (1, 2). CD8 acts as a coreceptor that can substantially enhance the sensitivity of antigen recognition in the context of low-affinity TCR/pMHCI interactions, whereas high-affinity TCR interactions are often sufficient to elicit activation without contribution from CD8 (3, 4).

CD8 is a glycoprotein that exists as a homodimer (CD8αα) or a heterodimer (CD8αβ). CD8^+^ T cells predominantly express CD8αβ, which is a more effective coreceptor than CD8αα (5, 6, 7). CD8αβ binds to a largely invariant region of the pMHCI complex at a site distinct from the TCR and stabilizes the TCR/pMHCI interaction (8, 9). The pMHCI/CD8 interaction is characterized by low solution binding affinities (mean K_D_ = 145 μM for CD8αα) (10), enabling antigen specificity to be conferred by the TCR and tuned by CD8 (11). This latter function is mediated distinctly by CD8α, which interacts with p56^lck^ to facilitate antigen-driven signal transduction directly (12, 13), and CD8β, which contains palmitoylation sites that promote colocalization of the TCR complex with kinase-rich areas of the cell membrane to facilitate antigen-driven signal transduction indirectly (7, 14).

The biological role of CD8 has been investigated extensively using panels of MHCI mutations that alter the strength of the pMHCI/CD8 interaction (11, 12, 15, 16, 17). These studies clearly showed that decreasing the affinity of the pMHCI/CD8 interaction impaired antigen sensitivity (11, 12) and that increasing the affinity of the pMHCI/CD8 interaction enhanced antigen sensitivity (15). Affinity increases beyond a certain threshold nonetheless resulted in non-specific antigen recognition (16, 17). These observations led to the concept of a pMHCI/CD8 therapeutic affinity window (17). Accordingly, it was predicted that engineered CD8 variants with supraphysiological affinities for the pMHCI complex below the threshold for non-specific activation, nominally ∼K_D_ >27 μM, would enhance the sensitivity of antigen recognition *via* low-affinity TCRs. In the present study, we tested this notion experimentally using model systems and further investigated the translational feasibility of such an approach using primary human CD4^+^ and CD8^+^ T cells transduced with clinically relevant cancer-targeting TCRs.

## Results

### Design of high-affinity CD8 variants

**Figure 1:**
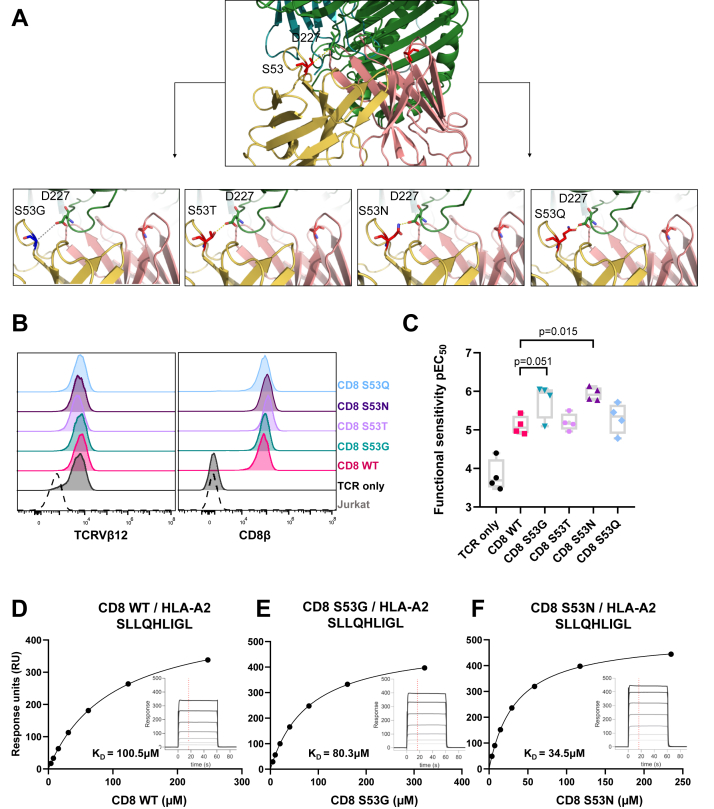
**Design and characterization of CD8 variants.***A*, crystal structure of human CD8αα complexed with HLA-A∗0201 (18) in cartoon form showing CD8α chain residue Ser53 (*red*) interacting with HLAα3 residue Asp227 (*green*). CD8α2 residue Ser53 mutations are shown in the four images below interacting with HLAα3 residue Asp227 (*green*). *B*, expression of TCRVβ12 and CD8β on Jurkat cells transduced with the RLA TCR and CD8αβ containing either wild-type (WT) CD8α or mutated forms (S53G, S53T, S53N, or S53Q) of CD8α. *C*, functional sensitivity of RLA TCR^+^ CD8αβ^+^ Jurkat cells expressed as the decimal cologarithm of the half-maximal efficacy concentration (pEC_50_). The activation of RLA TCR^+^ CD8αβ^+^ Jurkat cells in response to C1R HLA-A2 cells pulsed with serial dilutions of the cognate peptide was assessed by measuring the upregulation of CD69. Significance was determined using a one-way ANOVA with Dunnett’s *post hoc* test to compare each variant *versus* wild-type CD8 (n = 4). Data are derived from four separate experiments. *D*–*F*, representative surface plasmon resonance affinity measurements of wild-type (WT) CD8αα (*D*) and the most functionally potent variants of CD8αα, namely S53G (*E*) and S53N (*F*), *versus* SLL/HLA-A∗0201.

To predict candidate mutations that might enhance the strength of the pMHCI/CD8 interaction without exceeding the affinity threshold for non-specific activation, we focused on previously defined interaction points between the human leukocyte antigen (HLA) heavy chain α3 domain (Asp223–Asp227) and CD8α (Ser53) at the binding interface between HLA-A∗0201 (HLA-A2) and CD8αα (18) (Fig. 1*A*). The crystal structure of murine CD8αβ in complex with H-2D^d^ suggests that the CD8α1 chain in the homodimer is replaced with the CD8β chain in the heterodimer (19), which makes only Ser53 in the human CD8α2 chain relevant to the interaction with HLAα3. Alongside visual inspection of the interface using PyMOL software, the BUDE Alanine Scan (BAlaS) web tool was used to direct the mutagenesis strategy (20, 21). Using this approach as a guide, we replaced Ser53 (counting positionally from the first amino acid after the CD8 leader sequence) with amino acids bearing polar side-chains of increasing size, namely threonine (S53T), asparagine (S53N), and glutamine (S53Q), thereby progressively reducing the interaction distance between the corresponding side-chains and the HLA-A2α3 domain residue Asp227. In addition, we replaced Ser53 with glycine (S53G), an amino acid with the simplest side-chain, with the intention of abolishing the site-specific interaction between CD8α and the HLA-A2α3 domain residue Asp227 (Fig. 1*A*).

### Functional and biophysical analysis of CD8 variants

To determine if any of the CD8 variants described above improved antigen sensitivity, we introduced CD8αβ containing either wild-type or mutated forms (S53G, S53T, S53N, or S53Q) of the CD8α chain into Jurkat cells alongside an HLA-A2-restricted TCR specific for the RLARLALVL epitope (RLA TCR) derived from the cancer-associated antigen 5T4 (residues 18–25). RLA TCR^+^ CD8αβ^+^ Jurkat cells were purified *via* fluorescence-activated cell sorting (FACS) to express comparable levels of TCR and CD8β (Fig. 1*B*). It was notable in this context that the S53Q variant impacted the intensity of staining with antibodies directed against CD8α (clone RPA-T8) (Fig. S1*A*) and that the S53Q and S53T variants impacted the intensity of staining with antibodies directed against CD8αβ (clone 2ST8.5H7) (Fig. S1*B*). The activation of RLA TCR^+^ CD8αβ^+^ Jurkat cells in response to C1R HLA-A2 cells pulsed with serial dilutions of the cognate peptide was then assessed by measuring the upregulation of CD69, expressing functional sensitivity as the decimal cologarithm of the half-maximal efficacy concentration (pEC_50_). In direct comparisons with wild-type CD8, incorporation of the S53N variant improved the antigen sensitivity of RLA TCR^+^ CD8αβ^+^ Jurkat cells by almost an order of magnitude, and counterintuitively, a similar effect was observed with S53G (Fig. 1*C*).

To probe the mechanistic basis of these observations, we used surface plasmon resonance to measure the binding affinities of the CD8αα variants S53G and S53N for HLA-A2 refolded around three different clinically relevant peptide epitopes, namely preferentially expressed antigen of melanoma (PRAME)_425–433_ SLLQHLIGL (SLL), PRAME_100–108_ VLDGLDVLL (VLD), and Wilms’ tumor protein 1 (WT1)_126–134_ RMFPNAPYL (RMF). In line with the functional data, the CD8αα variants bound SLL/HLA-A2 with higher equilibrium affinities (S53G, K_D_ = 80.3 μM; S53N, K_D_ = 34.5 μM) than wild-type CD8αα (K_D_ = 100.5 μM) (Table 1, Fig. 1, *D*–*F*). Similar results were obtained with VLD/HLA-A2 (Table 1, Fig. S1*C*), and despite a lower overall affinity range, similar patterns were observed with RMF/HLA-A2 (Table 1, Fig. S1*D*).

**Table 1:** Surface plasmon resonance affinity measurements for wild-type (WT) CD8αα and the CD8αα variants S53G and S53N *versus* SLL/HLA-A∗0201, VLD/HLA-A∗0201, and RMF/HLA-A∗0201

CD8αα	HLA-A∗0201 SLLQHLIGL	HLA-A∗0201 VLDGLDVLL	HLA-A∗0201 RMFPNAPYL	Average K_D_
CD8αα WT	K_D_ = 100.5 μM	K_D_ = 99.2 μM	K_D_ = 193.0 μM	K_D_ = 131 μM
CD8αα S53G	K_D_ = 80.3 μM	K_D_ = 77.6 μM	K_D_ = 171.0 μM	K_D_ = 109.6 μM
CD8αα S53N	K_D_ = 34.5 μM	K_D_ = 34.0 μM	K_D_ = 76.3 μM	K_D_ = 48.2 μM

### High-affinity CD8 variants enhance the sensitivity of antigen recognition via low-affinity TCRs

Mathematical modeling predicts that the scope for enhancement by the CD8 coreceptor varies widely across different ligands due to non-linear relationships between kinetic parameters and functional sensitivity, such that near-optimal antigen recognition in the absence of CD8 is only minimally augmented or even diminished by the pMHCI/CD8 interaction (22, 23, 24). The high-affinity CD8 variants S53G and S53N would therefore be expected to enhance the process of antigen recognition preferentially in the context of low-affinity TCRs. We tested this idea initially using an HLA-A2-restricted TCR specific for the heteroclitic ELAGIGILTV epitope (MEL5 TCR) derived from the melanoma-associated antigen Melan-A/MART-1 (residues 27–35). The equilibrium binding affinities of the MEL5 TCR for the cognate epitope (ELA, K_D_ = 17 μM) and various altered peptide ligands (APLs), including ELTGIGILTV (3T, K_D_ = 82 μM) and FATGIGIITV (FAT, K_D_ = 3 μM), were reported previously (25, 26).

**Figure 2:**
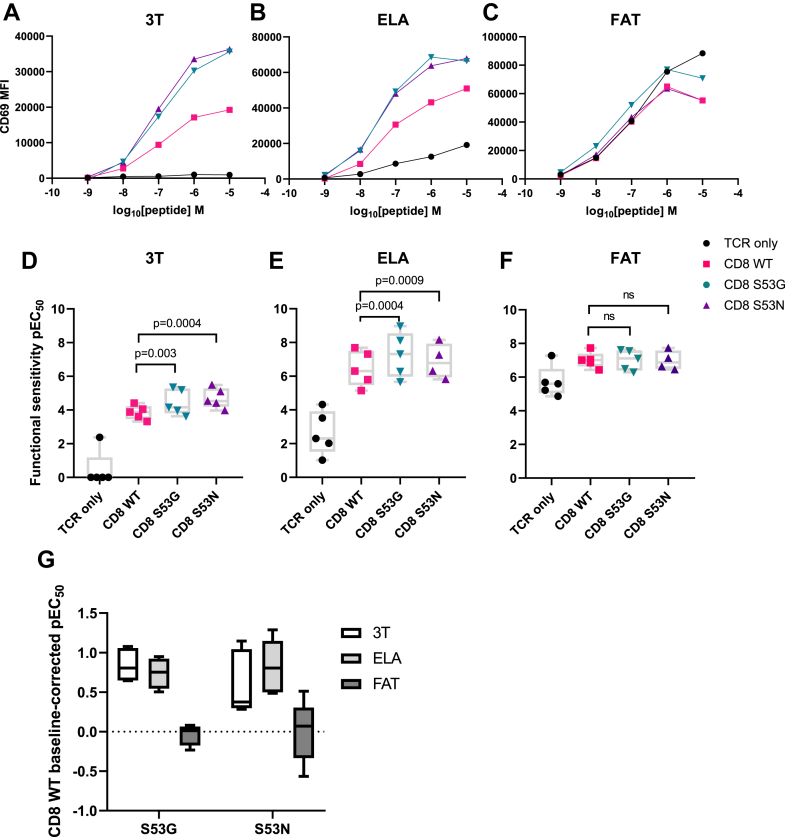
**High-affinity CD8 variants enhance the sensitivity of antigen recognition *via* low-affinity TCRs.***A*–*C*, representative titration curves showing the activation of MEL5 TCR^+^ CD8αβ^+^ JRT3-T3.5 cells in response to C1R HLA-A2 cells pulsed with serial dilutions of the 3T (*A*), ELA (*B*), or FAT peptides (*C*) assessed by measuring the upregulation of CD69. MEL5 TCR^+^ CD8αβ^+^ JRT3-T3.5 cells were transduced with CD8αβ containing either wild-type (WT) CD8α (*red*) or mutated forms of CD8α, namely S53G (*teal*) or S53N (*purple*). *D*–*F*, functional sensitivity of MEL5 TCR^+^ CD8αβ^+^ JRT3-T3.5 cells expressed as the decimal cologarithm of the half-maximal efficacy concentration (pEC_50_) for each of the conditions shown in *A*–*C*. Significance was determined using a one-way ANOVA with Dunnett’s *post hoc* test to compare each variant *versus* wild-type CD8 (n = 5). Data are derived from five separate experiments. *G*, data summary shown as baseline-corrected pEC_50_ values relative to wild-type CD8. MFI, geometric mean fluorescence intensity.

For the purposes of this study, we sequentially transduced the TCRβ chain-deficient JRT3-T3.5 cell line with the MEL5 TCR and CD8αβ containing either wild-type or mutated forms (S53G or S53N) of the CD8α chain and purified MEL5 TCR^+^ CD8αβ^+^ JRT3-T3.5 cells *via* FACS to express comparable levels of TCR and CD8β (Fig. S1, *E*–*H*). It was again notable that the S53N variant impacted the intensity of staining with antibodies directed against CD8αβ (clone 2ST8.5H7) (Fig. S1*G*), akin to S53Q and S53T. The activation of MEL5 TCR^+^ CD8αβ^+^ JRT3-T3.5 cells in response to C1R HLA-A2 cells pulsed with serial dilutions of the 3T, ELA, or FAT peptides was then assessed by measuring the upregulation of CD69. In direct comparisons with wild-type CD8, incorporation of the S53G or S53N variants improved the antigen sensitivity of MEL5 TCR^+^ CD8αβ^+^ JRT3-T3.5 cells for the low-affinity ligand 3T and the intermediate-affinity ligand ELA but not for the high-affinity ligand FAT (Fig. 2, *A*–*G*). These findings aligned with the data obtained using the RLA TCR, which exhibited a relatively low equilibrium binding affinity for RLA/HLA-A2 (K_D_ = 45 μM) (Fig. S2, *A* and *B*).

### High-affinity CD8 variants enhance the functional recognition of cancer cells via clinically relevant TCRs

To probe the biological relevance of these findings in the context of physiological antigen presentation, we introduced CD8αβ containing either wild-type or mutated forms (S53G or S53N) of the CD8α chain into a JE6.1 reporter cell line alongside either the HLA-A2-restricted 1E9 TCR or the HLA-A2-restricted KL14 TCR. This reporter system was designed to enable sensitive measurements of TCR-mediated activation *via* NFAT-driven expression of cyan fluorescent protein (CFP) and/or NFκB-driven expression of green fluorescent protein (GFP) (27, 28). The 1E9 TCR was isolated from an allogeneic HLA-A2-restricted repertoire specific for the SLFLGILSV epitope (SLF) derived from the B cell lineage marker CD20 (residues 188–196) (29), and the KL14 TCR was isolated from an autologous HLA-A2-restricted repertoire specific for the SLLMWITQC epitope (SLL) derived from the cancer-testis antigen CTAG1/NY-ESO-1 (residues 157–165) (30). Although the corresponding solution affinities remain unknown, the 1E9 TCR has been characterized as relatively antigen insensitive, preferentially recognizing target cell lines that express high levels of CD20 (29, 31), and likewise, the KL14 TCR has been characterized as relatively antigen insensitive, requiring high exogenous concentrations of the cognate peptide to mediate optimal functional responses against target cell lines displaying HLA-A2 (30).

**Figure 3:**
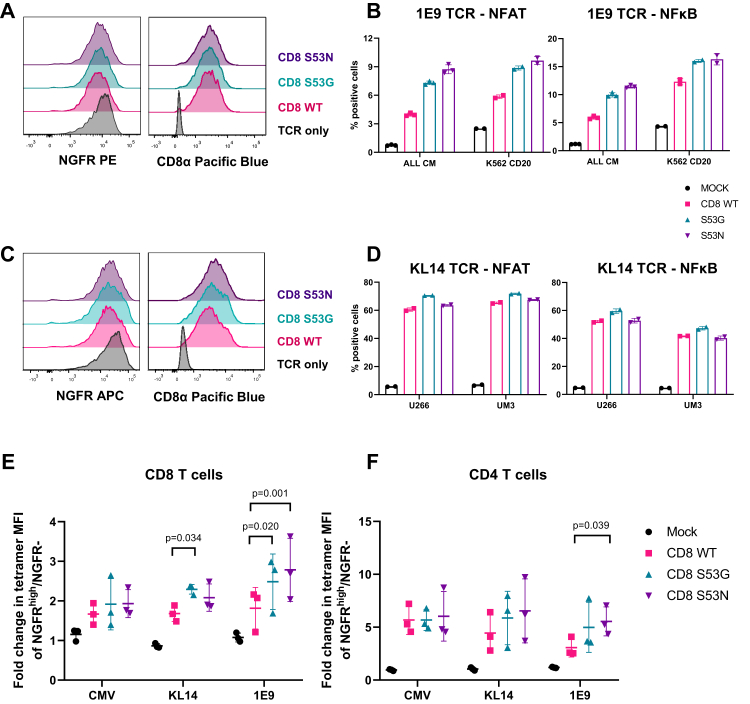
**High-affinity CD8 variants enhance signal transduction and tetramer uptake by cancer-targeting TCRs.***A*, expression of NGFR and CD8α among 1E9 TCR^+^ CD8αβ^+^ JE6.1 reporter cells transduced with CD8αβ containing either wild-type (WT) CD8α (*red*) or mutated forms of CD8α, namely S53G (*teal*) or S53N (*purple*). *B*, NFAT and NFκB reporter activity among the corresponding reporter cells in response to coculture with ALL CM cells or K562 HLA-A2+CD20 cells. *C*, expression of NGFR and CD8α among KL14 TCR^+^ CD8αβ^+^ JE6.1 reporter cells transduced with CD8αβ containing either wild-type (WT) CD8α (*red*) or mutated forms of CD8α, namely S53G (*teal*) or S53N (*purple*). *D*, NFAT and NFκB reporter activity among the corresponding reporter cells in response to coculture with UM3 cells or U266 cells. Data are shown as mean ± SD of duplicate or triplicate measurements from one experiment in (*B*) and (*D*). *E* and *F*, fold change in geometric mean fluorescence intensity (MFI) of cognate tetramer staining among NGFR^high^/NGFR^−^ populations of primary CD8^+^ T cells (*E*) or primary CD4^+^ T cells (*F*) transduced with the CMV TCR, the 1E9 TCR, or the KL14 TCR and CD8αβ containing either wild-type (WT) CD8α (*red*) or mutated forms of CD8α, namely S53G (*teal*) or S53N (*purple*). Data from three donors are shown as mean ± SD (*E* and *F*). Significance was determined using a one-way ANOVA with Dunnett’s *post hoc* test to compare each variant *versus* wild-type CD8.

1E9 TCR^+^ CD8αβ^+^ JE6.1 reporter cells and KL14 TCR^+^ CD8αβ^+^ JE6.1 reporter cells were purified *via* FACS to express comparable levels of truncated nerve growth factor receptor (NGFR), a downstream expression marker in the retroviral construct, and CD8α (Fig. 3, *A* and *C*). The activation of 1E9 TCR^+^ CD8αβ^+^ JE6.1 reporter cells in response to targets expressing CD20 (ALL CM and K562 HLA-A2+CD20) and KL14 TCR^+^ CD8αβ^+^ JE6.1 reporter cells in response to targets expressing CTAG1 (UM3 and U266) was assessed by measuring the upregulation of CFP and GFP (Fig. 3, *B* and *D*). In direct comparisons with wild-type CD8, incorporation of the S53G or S53N variants enhanced the activation of 1E9 TCR^+^ CD8αβ^+^ JE6.1 reporter cells cocultured with target cell lines expressing CD20 (Fig. 3*B*), whereas only marginal differences in activation were observed among KL14 TCR^+^ CD8αβ^+^ JE6.1 reporter cells cocultured with target cell lines expressing CTAG1 (Fig. 3*D*).

### High-affinity CD8 variants enhance tetramer binding to primary CD4^+^ and CD8^+^ T cells transduced with cognate TCRs

In further experiments, we isolated peripheral blood mononuclear cells (PBMCs) from three healthy adult donors and purified CD4^+^ and CD8^+^ T cells *via* magnetic separation. We then transduced these lineage-defined subsets with the 1E9 TCR, the KL14 TCR, or an HLA-A2-restricted TCR specific for the NLVPMVATV epitope (CMV TCR) derived from the human cytomegalovirus (HCMV) protein pp65 (residues 495–503), which exhibits a relatively high equilibrium binding affinity for NLV/HLA-A2 (K_D_ = 6.3 µM at 25°C) (32). Transduced CD4^+^ and CD8^+^ T cells were enriched *via* magnetic separation based on expression levels of the murine TCRβ constant region (mTCR) and subsequently transduced with vectors encoding CD8αβ containing either wild-type or mutated forms (S53G or S53N) of the CD8α chain alongside the expression marker NGFR. The uptake of fluorescent tetrameric antigens from solution was measured *via* flow cytometry and expressed as the fold change in geometric mean fluorescence intensity among cells expressing high levels of NGFR *versus* cells lacking expression of NGFR.

The high-affinity variants S53G and S53N only marginally enhanced tetramer staining among CD8^+^ T cells expressing the CMV TCR relative to wild-type CD8. In contrast, the S53G variant enhanced tetramer staining among CD8^+^ T cells expressing the KL14 TCR relative to wild-type CD8, and the S53G and S53N variants both enhanced tetramer staining among CD8^+^ T cells expressing the 1E9 TCR relative to wild-type CD8 (Fig. 3*E*). All three coreceptor constructs enhanced tetramer staining among CD4^+^ T cells, which lack endogenous CD8. The S53N variant also enhanced tetramer staining among CD4^+^ T cells expressing the 1E9 TCR relative to wild-type CD8 (Fig. 3*F*).

### Overexpression of CD8 enhances the functionality of primary CD8^+^ T cells transduced with cancer-targeting TCRs

To extend these findings, we examined the effector functions of primary CD8^+^ T cells transduced as above with CD8αβ containing either wild-type or mutated forms (S53G or S53N) of the CD8α chain alongside the 1E9 TCR, which has been shown to exhibit relatively weak recognition of target cell lines expressing low levels of CD20 (29, 31). Transduced cells were purified *via* FACS to express high levels of NGFR, thereby maximizing the expression ratio of exogenous *versus* endogenous CD8 (Fig. S3*A*). The introduction of either wild-type or mutated CD8 enhanced the production of interferon (IFN)-γ by 1E9 TCR-transduced primary CD8^+^ T cells cocultured with K562 HLA-A2+CD20 cells but not with K562 HLA-A2 cells, indicating an effect specific for CD20 (Fig. S3*B*). Similarly, the introduction of either wild-type or mutated CD8 enhanced the production of IFN-γ by KL14 TCR-transduced primary CD8^+^ T cells cocultured with UM3 cells, U266 cells, or Raji HLA-A2+CTAG1 cells but not with Raji HLA-A2 cells, indicating an effect specific for CTAG1 (Fig. S3*C*). No differences in the magnitude of these effects were observed among the different forms of CD8 (Fig. S3, *B* and *C*).

### High-affinity CD8 variants enhance the functionality of primary CD4^+^ T cells transduced with cancer-targeting TCRs

**Figure 4:**
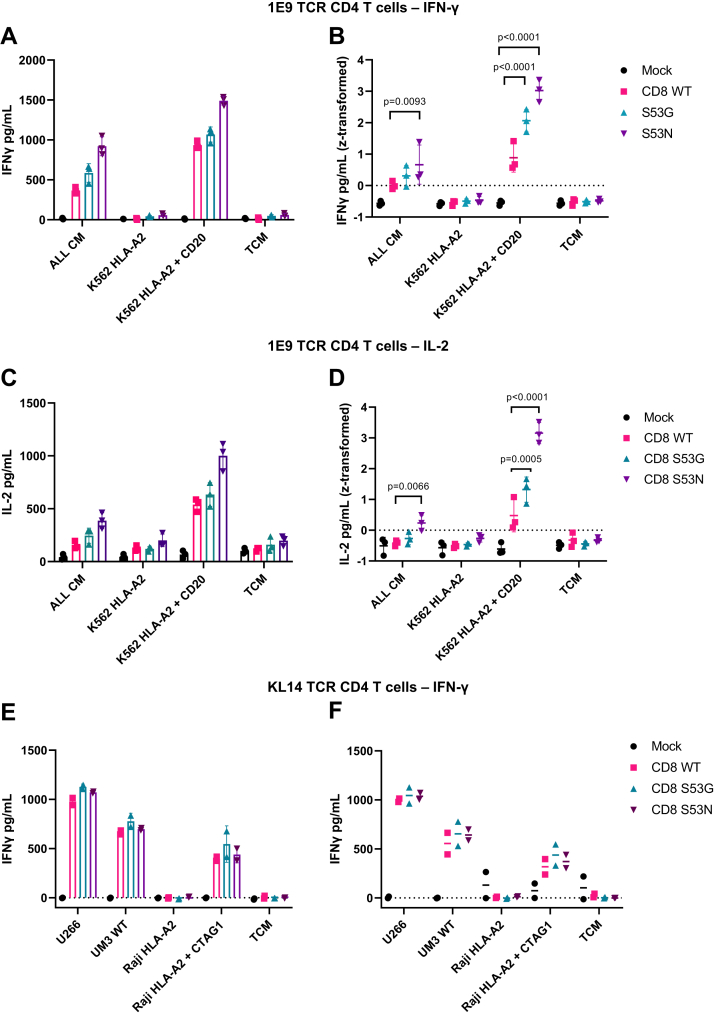
**High-affinity CD8 variants enhance the functionality of primary CD4**^**+**^**T cells transduced with cancer-targeting TCRs.***A–D*, primary CD4^+^ T cells expressing CD8αβ containing either wild-type (WT) CD8α (*red*) or mutated forms of CD8α, namely S53G (*teal*) or S53N (*purple*), alongside the 1E9 TCR were cocultured with a panel of cell lines lacking or expressing CD20. The panels show representative IFN-γ (*A*) or IL-2 production (*C*) from a single donor (triplicate measurements) or IFN-γ (*B*) or IL-2 production (*D*) from each of three donors. *E* and *F*, primary CD4^+^ T cells expressing CD8αβ containing either wild-type (WT) CD8α (*red*) or mutated forms of CD8α, namely S53G (*teal*) or S53N (*purple*), alongside the KL14 TCR were cocultured with a panel of cell lines lacking or expressing CTAG1. The panels show representative IFN-γ production (*E*) from a single donor (duplicate measurements) or IFN-γ production (*F*) from each of two donors. Data are shown as mean ± SD (*A*–*F*). Significance was determined using a one-way ANOVA with Dunnett’s *post hoc* test to compare each variant *versus* wild-type CD8. TCM, T cell medium.

In light of these results, we performed similar experiments with primary CD4^+^ T cells, thereby eliminating the potentially confounding effects of competition between exogenous and endogenous CD8. Primary CD4^+^ T cells expressing CD8αβ containing either wild-type or mutated forms (S53G or S53N) of the CD8α chain alongside the 1E9 TCR were generated as described above and purified *via* FACS to express high levels of NGFR and CD8α (Fig. S3*D*). In direct comparisons with wild-type CD8, incorporation of the S53G or S53N variants enhanced the production of IFN-γ by 1E9 TCR-transduced primary CD4^+^ T cells cocultured with K562 HLA-A2+CD20 cells but not with K562 HLA-A2 cells, and incorporation of the S53N variant enhanced the production of IFN-γ by 1E9 TCR-transduced primary CD4^+^ T cells cocultured with ALL CM cells, consistent with an effect specific for CD20 (Fig. 4, *A* and *B*). Similar effects were observed using other readouts of activation, namely the production of interleukin (IL)-2 (3/3 donors; Fig. 4, *C* and *D*) and proliferation (2/3 donors; Fig. S3*E*). The introduction of either wild-type or mutated CD8 also enhanced the production of IFN-γ by KL14 TCR-transduced primary CD4^+^ T cells cocultured with UM3 cells, U266 cells, or Raji HLA-A2+CTAG1 cells but not with Raji HLA-A2 cells, indicating an effect specific for CTAG1 that was nonetheless comparable among the different forms of CD8 (Fig. 4, *E* and *F*).

Collectively, these data showed that high-affinity variants of CD8, designed and engineered to remain below the equilibrium binding threshold for non-specific activation, selectively enhanced the functional recognition of clinically relevant pMHCI antigens *via* low-affinity TCRs.

## Discussion

Mutagenesis studies focused on key contact points in the MHCIα3 domain have demonstrated a critical role for CD8 in the process of TCR-mediated pMHCI antigen recognition (11, 12, 15, 16, 17, 33). In contrast, relatively few studies have addressed the impact of analogous mutations in CD8, at least in human systems, which limits the utility of such information for the purposes of translational immunotherapy (34, 35). We addressed this knowledge gap by designing and testing a panel of high-affinity CD8 variants biophysically and functionally to optimize the sensitivity of pMHCI antigen recognition in the context of clinically relevant TCRs.

A relatively small increase in the affinity of the pMHCI/CD8 interaction was afforded by the CD8α variant S53G (K_D_ = 80.3 μM), which nonetheless improved the antigen sensitivity of low-affinity TCRs in cotransduction experiments relative to wild-type CD8, consistent with data generated using reciprocal mutations in the MHCIα3 domain (15). This effect was further enhanced by the CD8α variant S53N, which afforded a greater increase in the affinity of the pMHCI/CD8 interaction (K_D_ = 34.5 μM), akin to a previously described compound mutation (C33A/S53N) (36). The ability of the S53G variant to enhance the affinity of the pMHCI/CD8 interaction was initially counterintuitive, because it had been designed to remove the interacting side-chain of Ser53. However, the difference in equilibrium binding affinity was only ∼20 μM relative to wild-type CD8, which equates to ∼0.5 kJ M^−1^, making accurate prediction almost impossible. It was also apparent that the side-chain of Ser53 slightly destabilized the pMHCI/CD8 complex, likely due to a combination of conformational entropy and strain effects and a rebalancing of entropy/enthalpy compensation. Accordingly, removal of this side-chain potentially enabled flexibility in the protein backbone, facilitating a conformational change that strengthened the interaction with pMHCI. It is notable that our biophysical data were generated using soluble CD8αα, whereas the functional experiments were performed using transduced CD8αβ. However, the biophysical measurements reported here are likely extendable, given that murine CD8αα and murine CD8αβ bind with similar affinities to pMHCI (10).

Our experiments with APLs showed that high-affinity CD8 variants improved the recognition of weak agonists but not optimal agonists, at least in the context of the MEL5 TCR. A generic tuning effect that preferentially enhances the recognition of affinity-limited pMHCI antigens would potentially be very useful in the setting of cancer immunotherapy. Most cancer-associated antigens are derived from self-proteins, which shape thymic selection, leading to the deletion of high-affinity cognate TCRs. As a consequence, cancer-associated antigen recognition in the periphery is dominated by low-affinity TCRs. The use of high-affinity CD8 variants could bypass this limitation without incurring unpredictable off-target reactivity, which is an inherent risk of alternative strategies based on affinity maturation (37, 38, 39).

We explored the potential utility of this approach in primary CD4^+^ and CD8^+^ T cells transduced with clinically relevant TCRs targeting either CD20 or CTAG1 (NY-ESO1). The introduction of either wild-type or high-affinity CD8 variants into primary CD8^+^ T cells enhanced the sensitivity of antigen recognition to a similar degree, suggesting that coreceptor expression levels were a key determinant of activation (40, 41). The observation that the high-affinity CD8 variants afforded little improvement beyond that achieved using wild-type CD8 could be explained by competition with endogenous CD8. Strategies designed to knock down endogenous CD8 could offer a solution to this problem, although it might be difficult to match natural expression levels using viral delivery. Alternatively, a CRISPR-Cas9 homology-directed repair strategy incorporating point mutations could be a feasible option, ideally in conjunction with a molecular tag for bulk verification (42).

High-affinity CD8 variants could also be introduced into primary CD4^+^ T cells, which have been shown to be effective vehicles in the setting of cancer immunotherapy. In our experiments, this approach revealed the potential of high-affinity CD8 variants to enhance antigen sensitivity, although the magnitude of this effect appeared to depend on the cotransduced TCR. Differences in the nature of antigen engagement likely underpinned this observation, but further studies will be required to define the precise biophysical and structural determinants of an optimal response (3, 4). TCR-transduced CD4^+^ and CD8^+^ T cells are already being used in clinical trials (43, 44, 45), some of which have also been engineered to coexpress CD8 (NCT04044859, NCT03326921). The use of high-affinity CD8 variants is eminently compatible with such approaches and could further enhance the efficacy of protocols in which bulk CD3^+^ T cells are transduced to express cancer-targeting TCRs.

Xenograft models of hematological malignancies and solid tumors are generally used to evaluate novel therapeutic strategies but provide limited information regarding the safety of any particular intervention (38, 39). Syngeneic mouse models are similarly limited in terms of applicability, because murine CD8 binds with a higher affinity to pMHCI (K_D_ = 49 μM) than human CD8 (K_D_ = 145 μM). However, it will be essential to examine the potential off-target effects associated with the use of high-affinity CD8 variants prior to translation, especially given the preferential enhancement of antigen recognition in the context of low-affinity TCR/pMHCI interactions, which could feasibly induce autoimmunity (24). We observed no such effects in the absence of cognate antigen, but *in vitro* screens for reactivity against cell lines derived from healthy and malignant tissues combined with the use of combinatorial peptide libraries to identify relevant autoantigens would be required at a minimum to exclude this possibility (39, 46, 47).

TCR engineering is a rapidly emerging approach to cancer immunotherapy (43, 44, 45). In this study, we characterized the biophysical and functional properties of two high-affinity CD8 variants, S53G and S53N, both of which operated within the therapeutic affinity window (17) to enhance the sensitivity of pMHCI antigen recognition in the context of low-affinity TCRs. Accordingly, our findings could provide a generically applicable strategy to augment the therapeutic efficacy of cancer-targeting TCRs, clinical trials of which have already started to incorporate wild-type CD8.

## Experimental procedures

### Ethics

This study was approved by the Institutional Review Board of the Leiden University Medical Center (3.4205/010/FB/jr) and the METC-LDD (HEM 008/SH/sh). Informed consent was obtained from all participants in accordance with the principles of the Declaration of Helsinki.

### Samples

PBMCs were isolated from healthy donors *via* standard density gradient centrifugation and cryopreserved at the Leiden University Medical Center Biobank for Hematological Diseases.

### Parental cell lines

C1R HLA-A2 cells, Jurkat cells, and J.RT3-T3.5 cells were cultured in RPMI medium supplemented with 10% fetal bovine serum (FBS), 1% penicillin/streptomycin, and 1% L-glutamine (Lonza). The generation of C1R HLA-A2 cells was described previously (15). JE6.1 Jurkat cells, K562 A2 cells, K562 HLA-A2+CD20 cells, Raji HLA-A2 cells, Raji HLA-A2+CTAG1 cells, and U266 cells were cultured in Iscove’s Modified Dulbecco’s Medium (IMDM) supplemented with 10% FBS, 1% penicillin/streptomycin, and 1.5% L-glutamine (Lonza). ALL CM cells were cultured in IMDM containing serum-free supplement and 1% penicillin/streptomycin as described previously (48). UM3 cells were cultured in IMDM supplemented with 20% FBS, 1% penicillin/streptomycin, 1.5% L-glutamine, and 10 ng/ml IL-6 (Lonza). HEK 293T cells were cultured in Dulbecco’s Modified Eagle Medium (DMEM) supplemented with 10% FBS, 1% penicillin/streptomycin, 1% L-glutamine, and 1 mM HEPES (Lonza). Phoenix Ampho cells were cultured in IMDM supplemented with 10% FBS, 1% penicillin/streptomycin, and 1% L-glutamine (Lonza).

### Soluble protein expression and surface plasmon resonance

CD8α chain sequences were codon-optimized for expression in *E. coli* and cloned into pGMT7. Point mutations for variant generation were introduced using a QuikChange II Site-Directed Mutagenesis Kit (Agilent). Soluble CD8αα was produced as described previously (36). CD8αα was concentrated after exchange into the running buffer (0.005% polysorbate 20 in PBS). Surface plasmon resonance experiments were performed using a CM5 sensor chip (GE Healthcare) activated *via* N-hydroxysuccinimide/1-ethyl-3-(3-dimethylaminopropyl)-1-carbodiimide hydrochloride (NHS/EDC) coupling with streptavidin (0.2 mg/ml in 10 mM acetate). The following biotinylated monomers were bound to the chip: D227K/T228A-A2-RLARLALVL, WT-A2-SLLMWITQC, WT-A2-VLDFAPPGA, and WT-A2-RMFPNAPYL. Serial dilutions of CD8αα were flowed over the chip at a rate of 10 μl/min. Response curves were aligned in time. Reference responses were subtracted using BIAEvaluation software (Biacore AB). Biophysical characterization of the RLA TCR was performed as described previously (3). TCRα and TCRβ sequences were obtained using a template-switch anchored RT-PCR (49).

### Design of point mutations in CD8α

The crystal structure of HLA-A∗0201 in complex with CD8αα (PDB ID 1AKJ) was uploaded to the BAlaS web tool, an interactive application linked with the Bristol University Docking Engine (BUDE) Alanine Scan (BudeAlaScan) (20, 21). The two CD8α chains were selected as ligands, the HLA-A∗0201 α1, α2, and α3 domains and β2-microglobulin were selected as receptors, and the mutagenesis strategy was informed by outputs listed as the predicted difference in binding affinity for each amino acid change (ΔΔG).

### Viral vector constructs and virus production

Lentiviral vectors encoding CD8αβ were described previously (24). Point mutations yielding the CD8 variants S53T, S53Q, S53N, and S53G were introduced into lentiviral vectors using a Q5 Site-Directed Mutagenesis Kit (New England Biolabs). For retroviral expression, the CD8α and CD8β chains, separated by a P2A cleavage site (Genewiz), were cloned into an MP71 vector containing an IRES sequence and a downstream expression marker (truncated NGFR). The TCRα and TCRβ chains of the RLA TCR were partly murinized (50) and cloned into pSF.EF1α. The MEL5 TCR lentiviral construct was described previously (24). The 1E9 TCR, the KL14 TCR, and the CMV TCR were partly murinized and cloned into MP71 (29). Lentiviral particles were produced in HEK 293T cells *via* cotransfection of the relevant pSF.EF1α plasmid with pMDL/pRRE, pRSV-Rev, and pCMV.VSV-G using Turbofect Transfection Reagent (Thermo Fisher Scientific). Supernatants were concentrated using Lenti-X Concentrator (Takara). Retroviral particles were produced similarly in Phoenix Ampho cells *via* cotransfection of the relevant MP71 plasmid with pCL-Ampho.

### Jurkat cell lines

MEL5 TCR^+^ CD8^+^ J.RT3-T3.5 cells were described previously (24). Jurkat cells expressing the RLA TCR and variant or wild-type CD8 were generated similarly and standardized for comparable expression levels *via* FACS. JE6.1 Jurkat cells were described previously (27). Reporter cells expressing comparable levels of the 1E9 TCR or the KL14 TCR and variant or wild-type CD8 were generated *via* cotransduction of JE6.1 Jurkat cells and subsequent purification *via* FACS.

### Transduction of primary human T cells

CD4^+^ and CD8^+^ T cells were isolated from thawed PBMCs *via* magnetic separation using CD4 and CD8 MicroBeads, respectively (Miltenyi Biotec). Freshly isolated cells were activated with phytohemagglutinin (0.8 μg/ml, Thermo Fisher Scientific) in the presence of irradiated autologous PBMCs and cultured for 48 h in IMDM supplemented with 5% human AB serum, 5% FBS, 1% penicillin/streptomycin, 1.5% L-glutamine (all from Lonza), and 100 IU/ml IL-2 (Novartis). Cells were then transduced with retroviral particles containing the 1E9 TCR, the KL14 TCR, or the CMV TCR *via* spinoculation on non-treated, retronectin-coated culture plates (Takara Bio). TCR-transduced cells were stained with anti-mouse TCRβ–APC (clone H57-597, BioLegend) and enriched using Anti-APC MicroBeads (Miltenyi Biotec). Enriched cells were cultured for 7 to 10 days, restimulated, and transduced with retroviral particles containing variant or wild-type CD8. TCR^+^ CD8^+^ populations were purified *via* FACS.

### Coculture experiments

C1R HLA-A2 and Jurkat cell coculture experiments were performed as described previously (24). Briefly, 1.5 × 10^5^ C1R HLA-A2 cells were pulsed with peptide for 1 h and cocultured with 3 × 10^4^ Jurkat cells for 6 h. Jurkat cell activation was measured using flow cytometry to quantify the expression of CD69. Similarly, 1.5 × 10^5^ target cells were cocultured with 3 × 10^4^ TCR^+^ CD8^+^ reporter Jurkat cells for 6 h. Jurkat cell activation was measured using flow cytometry to quantify the expression of CFP and GFP. Primary CD4^+^ or CD8^+^ T cells were rested for 24 to 48 h after purification *via* FACS. Aliquots of 1 × 10^3^ primary CD4^+^ or CD8^+^ T cells were then cocultured with 1 × 10^4^ irradiated ALL CM cells or 5 × 10^3^ irradiated K562 HLA-A2 cells, K562 HLA-A2+CD20 cells, Raji HLA-A2 cells, Raji HLA-A2+CTAG1 cells, UM3 cells, or U266 cells for 16 to 18 h in IMDM supplemented with 5% human AB serum, 5% FBS, and 1% penicillin/streptomycin (Lonza). Supernatants were collected and assayed using enzyme-linked immunosorbent assays to quantify IFN-γ and IL-2 (Diaclone). The cultures were then supplemented with fresh medium containing 50 IU/ml IL-2 (Novartis), incubated for a further 5 days, and resuspended in SYTOX Blue Dead Cell Stain (1:1,000, Thermo Fisher Scientific). Proliferation was measured using isovolumetric flow cytometry to quantify viable cells defined by the absence of SYTOX.

### Antibodies and peptides

The following antibodies were used in the study: (i) anti-human CD4–FITC (clone RPA-T4), anti-human CD69–BV421 (clone FN50), and anti-mouse TCRβ–PE or TCRβ–APC (clone H57-597) from BioLegend; (ii) anti-human CD8α–PE-Cy7 (clone 53-6.7) from Thermo Fisher Scientific; (iii) anti-human CD8β–eFluor 660 (clone SIDI8bee) from eBioscience; (iv) anti-human CD8β–PE (clone 2ST8.5H7) and anti-human NGFR–PE (clone C40-1457) from BD Biosciences; (v) anti-human TCR Vβ12.1–PE (clone VER2.32.1) from Beckman Coulter; and (vi) anti-human NGFR–APC (clone ME20.4) from Cedarlane. Staining was performed for 15 min at room temperature. All peptides were synthesized at >95% purity using standard Fmoc chemistry (BioSynthesis).

### Tetramers

The following tetramers were used in this study: HLA-A2-SLFLGILSV (CD20), HLA-A2-SLLMWITQC (CTAG1), and HLA-A2-NLVPMVATV (CMV). Each tetramer was used at a final concentration of 50 ng/ml. Staining was performed for 30 min at 37 °C.

### Flow cytometry

Data from Jurkat cells transduced with the MEL5 TCR or the RLA TCR were acquired using an ACEA NovoCyte (Agilent). Data from Jurkat reporter cells were acquired using a Fortessa (BD Biosciences). Data from primary cells were acquired using a Fortessa or an LSR II (BD Biosciences). Jurkat cells transduced with the MEL5 TCR or the RLA TCR were sorted using an Influx (BD Biosciences). Jurkat reporter cells and primary CD4^+^ or CD8^+^ T cells were sorted using a FACSAria (BD Biosciences).

### Statistics

Functional assay data were processed using simultaneous non-linear least square curve fitting and z-transformed where necessary to eliminate interdonor variability. Functional sensitivity was expressed as the decimal cologarithm of the half-maximal efficacy concentration (pEC_50_). Jurkat model system data were analyzed using a one-way ANOVA with Dunnett’s *post hoc* test to compare each variant *versus* wild-type CD8. Tetramer data were analyzed using a two-way ANOVA with Dunnett’s *post hoc* test. All statistical tests were performed using Prism (GraphPad).

## Data availability

All data are contained within the manuscript.

## Supporting information

This article contains supporting information.

## Conflict of interest

L. W., R. B. S., and L. K. have submitted an international patent application *Int Pat App WO 2022/112,752* on the basis of these results. All other authors declare no conflict of interest.

## References

[1] Aleksic M., Liddy N., Molloy P.E., Pumphrey N., Vuidepot A., Chang K.M. (2012). Different affinity windows for virus and cancer-specific T-cell receptors: implications for therapeutic strategies. Eur. J. Immunol..

[2] Bridgeman J.S., Sewell A.K., Miles J.J., Price D.A., Cole D.K. (2012). Structural and biophysical determinants of αβ T-cell antigen recognition. Immunology.

[3] Laugel B., van den Berg H.A., Gostick E., Cole D.K., Wooldridge L., Boulter J. (2007). Different T cell receptor affinity thresholds and CD8 coreceptor dependence govern cytotoxic T lymphocyte activation and tetramer binding properties. J. Biol. Chem..

[4] Holler P.D., Kranz D.M. (2003). Quantitative analysis of the contribution of TCR/pepMHC affinity and CD8 to T cell activation. Immunity.

[5] Crooks M.E., Littman D.R. (1994). Disruption of T lymphocyte positive and negative selection in mice lacking the CD8β chain. Immunity.

[6] Renard V., Romero P., Vivier E., Malissen B., Luescher I.F. (1996). CD8β increases CD8 coreceptor function and participation in TCR-ligand binding. J. Exp. Med..

[7] Arcaro A., Grégoire C., Boucheron N., Stotz S., Palmer E., Malissen B. (2000). Essential role of CD8 palmitoylation in CD8 coreceptor function. J. Immunol..

[8] Wooldridge L., van den Berg H.A., Glick M., Gostick E., Laugel B., Hutchinson S.L. (2005). Interaction between the CD8 coreceptor and major histocompatibility complex class I stabilizes T cell receptor-antigen complexes at the cell surface. J. Biol. Chem..

[9] Jiang N., Huang J., Edwards L.J., Liu B., Zhang Y., Beal C.D. (2011). Two-stage cooperative T cell receptor-peptide major histocompatibility complex-CD8 trimolecular interactions amplify antigen discrimination. Immunity.

[10] Cole D.K., Laugel B., Clement M., Price D.A., Wooldridge L., Sewell A.K. (2012). The molecular determinants of CD8 co-receptor function. Immunology.

[11] Hutchinson S.L., Wooldridge L., Tafuro S., Laugel B., Glick M., Boulter J.M. (2003). The CD8 T cell coreceptor exhibits disproportionate biological activity at extremely low binding affinities. J. Biol. Chem..

[12] Purbhoo M.A., Boulter J.M., Price D.A., Vuidepot A.L., Hourigan C.S., Dunbar P.R. (2001). The human CD8 coreceptor effects cytotoxic T cell activation and antigen sensitivity primarily by mediating complete phosphorylation of the T cell receptor ζ chain. J. Biol. Chem..

[13] Yamaguchi H., Hendrickson W.A. (1996). Structural basis for activation of human lymphocyte kinase Lck upon tyrosine phosphorylation. Nature.

[14] Doucey M.-A., Goffin L., Naeher D., Michielin O., Baumgärtner P., Guillaume P. (2003). CD3δ establishes a functional link between the T cell receptor and CD8. Immunity.

[15] Wooldridge L., Lissina A., Vernazza J., Gostick E., Laugel B., Hutchinson S.L. (2007). Enhanced immunogenicity of CTL antigens through mutation of the CD8 binding MHC class I invariant region. Eur. J. Immunol..

[16] Wooldridge L., Clement M., Lissina A., Edwards E.S.J., Ladell K., Ekeruche J. (2010). MHC class I molecules with superenhanced CD8 binding properties bypass the requirement for cognate TCR recognition and nonspecifically activate CTLs. J. Immunol..

[17] Dockree T., Holland C.J., Clement M., Ladell K., McLaren J.E., van den Berg H.A. (2017). CD8^+^ T-cell specificity is compromised at a defined MHCI/CD8 affinity threshold. Immunol. Cell Biol..

[18] Gao G.F., Tormo J., Gerth U.C., Wyer J.R., McMichael A.J., Stuart D.I. (1997). Crystal structure of the complex between human CD8αα and HLA-A2. Nature.

[19] Wang R., Natarajan K., Margulies D.H. (2009). Structural basis of the CD8αβ/MHC class I interaction: focused recognition orients CD8β to a T cell proximal position. J. Immunol..

[20] Wood C.W., Ibarra A.A., Bartlett G.J., Wilson A.J., Woolfson D.N., Sessions R.B. (2020). BAlaS: fast, interactive and accessible computational alanine-scanning using BudeAlaScan. Bioinformatics.

[21] Ibarra A.A., Bartlett G.J., Hegedüs Z., Dutt S., Hobor F., Horner K.A. (2019). Predicting and experimentally validating hot-spot residues at protein-protein interfaces. ACS Chem. Biol..

[22] Szomolay B., Williams T., Wooldridge L., van den Berg H.A. (2013). Co-receptor CD8-mediated modulation of T-cell receptor functional sensitivity and epitope recognition degeneracy. Front. Immunol..

[23] van den Berg H.A., Wooldridge L., Laugel B., Sewell A.K. (2007). Coreceptor CD8-driven modulation of T cell antigen receptor specificity. J. Theor. Biol..

[24] Clement M., Knezevic L., Dockree T., McLaren J.E., Ladell K., Miners K.L. (2021). CD8 coreceptor-mediated focusing can reorder the agonist hierarchy of peptide ligands recognized via the T cell receptor. Proc. Natl. Acad. Sci. U. S. A..

[25] Clement M., Ladell K., Ekeruche-Makinde J., Miles J.J., Edwards E.S.J., Dolton G. (2011). Anti-CD8 antibodies can trigger CD8^+^ T cell effector function in the absence of TCR engagement and improve peptide-MHCI tetramer staining. J. Immunol..

[26] Ekeruche-Makinde J., Clement M., Cole D.K., Edwards E.S.J., Ladell K., Miles J.J. (2012). T-cell receptor-optimized peptide skewing of the T-cell repertoire can enhance antigen targeting. J. Biol. Chem..

[27] Jutz S., Leitner J., Schmetterer K., Doel-Perez I., Majdic O., Grabmeier-Pfistershammer K. (2016). Assessment of costimulation and coinhibition in a triple parameter T cell reporter line: simultaneous measurement of NF-κB, NFAT and AP-1. J. Immunol. Methods.

[28] Rosskopf S., Leitner J., Paster W., Morton L.T., Hagedoorn R.S., Steinberger P. (2018). A Jurkat 76 based triple parameter reporter system to evaluate TCR functions and adoptive T cell strategies. Oncotarget.

[29] Jahn L., van der Steen D.M., Hagedoorn R.S., Hombrink P., Kester M.G.D., Schoonakker M.P. (2016). Generation of CD20-specific TCRs for TCR gene therapy of CD20^low^ B-cell malignancies insusceptible to CD20-targeting antibodies. Oncotarget.

[30] Roex M.C.J., Hageman L., Veld S.A.J., van Egmond E., Hoogstraten C., Stemberger C. (2020). A minority of T cells recognizing tumor-associated antigens presented in self-HLA can provoke antitumor reactivity. Blood.

[31] Wachsmann T.L.A., Wouters A.K., Remst D.F.G., Hagedoorn R.S., Meeuwsen M.H., van Diest E. (2022). Comparing CAR and TCR engineered T cell performance as a function of tumor cell exposure. Oncoimmunology.

[32] Gakamsky D.M., Lewitzki E., Grell E., Saulquin X., Malissen B., Montero-Julian F. (2007). Kinetic evidence for a ligand-binding-induced conformational transition in the T cell receptor. Proc. Natl. Acad. Sci. U. S. A..

[33] Wooldridge L., Laugel B., Ekeruche J., Clement M., van den Berg H.A., Price D.A. (2010). CD8 controls T cell cross-reactivity. J. Immunol..

[34] Cole D.K., Pumphrey N.J., Boulter J.M., Sami M., Bell J.I., Gostick E. (2007). Human TCR-binding affinity is governed by MHC class restriction. J. Immunol..

[35] Devine L., Thakral D., Nag S., Dobbins J., Hodsdon M.E., Kavathas P.B. (2006). Mapping the binding site on CD8β for MHC class I reveals mutants with enhanced binding. J. Immunol..

[36] Cole D.K., Rizkallah P.J., Boulter J.M., Sami M., Vuidepot A., Glick M. (2007). Computational design and crystal structure of an enhanced affinity mutant human CD8αα coreceptor. Proteins.

[37] Morgan R.A., Yang J.C., Kitano M., Dudley M.E., Laurencot C.M., Rosenberg S.A. (2010). Case report of a serious adverse event following the administration of T cells transduced with a chimeric antigen receptor recognizing ERBB2. Mol. Ther..

[38] Linette G.P., Stadtmauer E.A., Maus M.V., Rapoport A.P., Levine B.L., Emery L. (2013). Cardiovascular toxicity and titin cross-reactivity of affinity-enhanced T cells in myeloma and melanoma. Blood.

[39] Cameron B.J., Gerry A.B., Dukes J., Harper J.V., Kannan V., Bianchi F.C. (2013). Identification of a titin-derived HLA-A1-presented peptide as a cross-reactive target for engineered MAGE A3-directed T cells. Sci. Transl. Med..

[40] Bajwa G., Lanz I., Cardenas M., Brenner M.K., Arber C. (2020). Transgenic CD8αβ co-receptor rescues endogenous TCR function in TCR-transgenic virus-specific T cells. J. Immunother. Cancer.

[41] Rath J.A., Bajwa G., Carreres B., Hoyer E., Gruber I., Martínez-Paniagua M.A. (2020). Single-cell transcriptomics identifies multiple pathways underlying antitumor function of TCR- and CD8αβ-engineered human CD4^+^ T cells. Sci. Adv..

[42] Roth T.L., Puig-Saus C., Yu R., Shifrut E., Carnevale J., Li P.J. (2018). Reprogramming human T cell function and specificity with non-viral genome targeting. Nature.

[43] Dossa R.G., Cunningham T., Sommermeyer D., Medina-Rodriguez I., Biernacki M.A., Foster K. (2018). Development of T cell immunotherapy for hematopoietic stem cell transplantation recipients at risk of leukemia relapse. Blood.

[44] Nagarsheth N.B., Norberg S.M., Sinkoe A.L., Adhikary S., Meyer T.J., Lack J.B. (2021). TCR-engineered T cells targeting E7 for patients with metastatic HPV-associated epithelial cancers. Nat. Med..

[45] Rapoport A.P., Stadtmauer E.A., Binder-Scholl G.K., Goloubeva O., Vogl D.T., Lacey S.F. (2015). NY-ESO-1-specific TCR-engineered T cells mediate sustained antigen-specific antitumor effects in myeloma. Nat. Med..

[46] Bijen H.M., van der Steen D.M., Hagedoorn R.S., Wouters A.K., Wooldridge L., Falkenburg J.H.F. (2018). Preclinical strategies to identify off-target toxicity of high-affinity TCRs. Mol. Ther..

[47] Crowther M.D., Dolton G., Legut M., Caillaud M.E., Lloyd A., Attaf M. (2020). Genome-wide CRISPR–Cas9 screening reveals ubiquitous T cell cancer targeting via the monomorphic MHC class I-related protein MR1. Nat. Immunol..

[48] Nijmeijer B.A., Szuhai K., Goselink H.M., van Schie M.L.J., van der Burg M., de Jong D. (2009). Long-term culture of primary human lymphoblastic leukemia cells in the absence of serum or hematopoietic growth factors. Exp. Hematol..

[49] Quigley M.F., Almeida J.R., Price D.A., Douek D.C. (2011). Unbiased molecular analysis of T cell receptor expression using template-switch anchored RT-PCR.. Curr. Protoc. Immunol..

[50] Sommermeyer D., Uckert W. (2010). Minimal amino acid exchange in human TCR constant regions fosters improved function of TCR gene-modified T cells. J. Immunol..

