# Supplementary material for: High-affinity CD8 variants enhance the sensitivity of pMHCI antigen recognition via low-affinity TCRs
Source: J Biol Chem. 2023 Jun 28;299(8):104981. doi: 10.1016/j.jbc.2023.104981 (PMC10432799; doi:10.1016/j.jbc.2023.104981)
Supplement: Supporting Figures S1–S3 [file mmc1.pdf]

Supporting information

High-affinity CD8 variants enhance the sensitivity of pMHCI antigen recognition via low-affinity TCRs

Lea Knezevic[1,2*], Tassilo L. A. Wachsmann[2*], Ore Francis[1], Tamsin Dockree[3], John S. Bridgeman[4], Anne Wouters[2], Ben de Wet[5], David K. Cole[3,5], Mathew Clement[3,6], James E. McLaren[3], Emma Gostick[3], Kristin Ladell[3], Sian Llewellyn-Lacey[3], David A. Price[3,6], Hugo van den Berg[7], Zsuzsanna Tabi[3], Richard B. Sessions[8*], Mirjam H. M. Heemskerk[2*], Linda Wooldridge[1*]

[1]Faculty of Health Sciences, University of Bristol, Biomedical Sciences Building, Bristol, UK

[2]Department of Haematology, Leiden University Medical Center, Leiden, The Netherlands

[3]Division of Infection and Immunity, Cardiff University School of Medicine, University Hospital of Wales, Cardiff, UK

[4]Instil Bio, Inc., Dallas, TX

[5]Immunocore, Abingdon, UK

[6]Systems Immunity Research Institute, Cardiff University School of Medicine, University Hospital of Wales, Cardiff, UK

[7]Warwick Mathematics Institute, University of Warwick, Coventry, UK

[8]Faculty of Life Sciences, University of Bristol, Biomedical Sciences Building, Bristol, UK

*These authors contributed equally to this work.

Correspondence: Professor Linda Wooldridge, Faculty of Health Sciences, University of Bristol, Biomedical Sciences Building, Bristol BS8 1TD, UK. Email: linda.wooldridge@bristol.ac.uk.

The supporting information contains the following figures:

Figure S1 Biophysical and cellular characterization of CD8 variants.

Figure S2. Sequencing and biophysical characterization of the RLA TCR

Figure S3 Functional analysis of primary $CD4^+$ and $CD8^+$ T cells transduced with tumor-targeting TCRs.

S1 Phenotyping of transgenic Jurkat cells and affinity measurements of CD8 variants

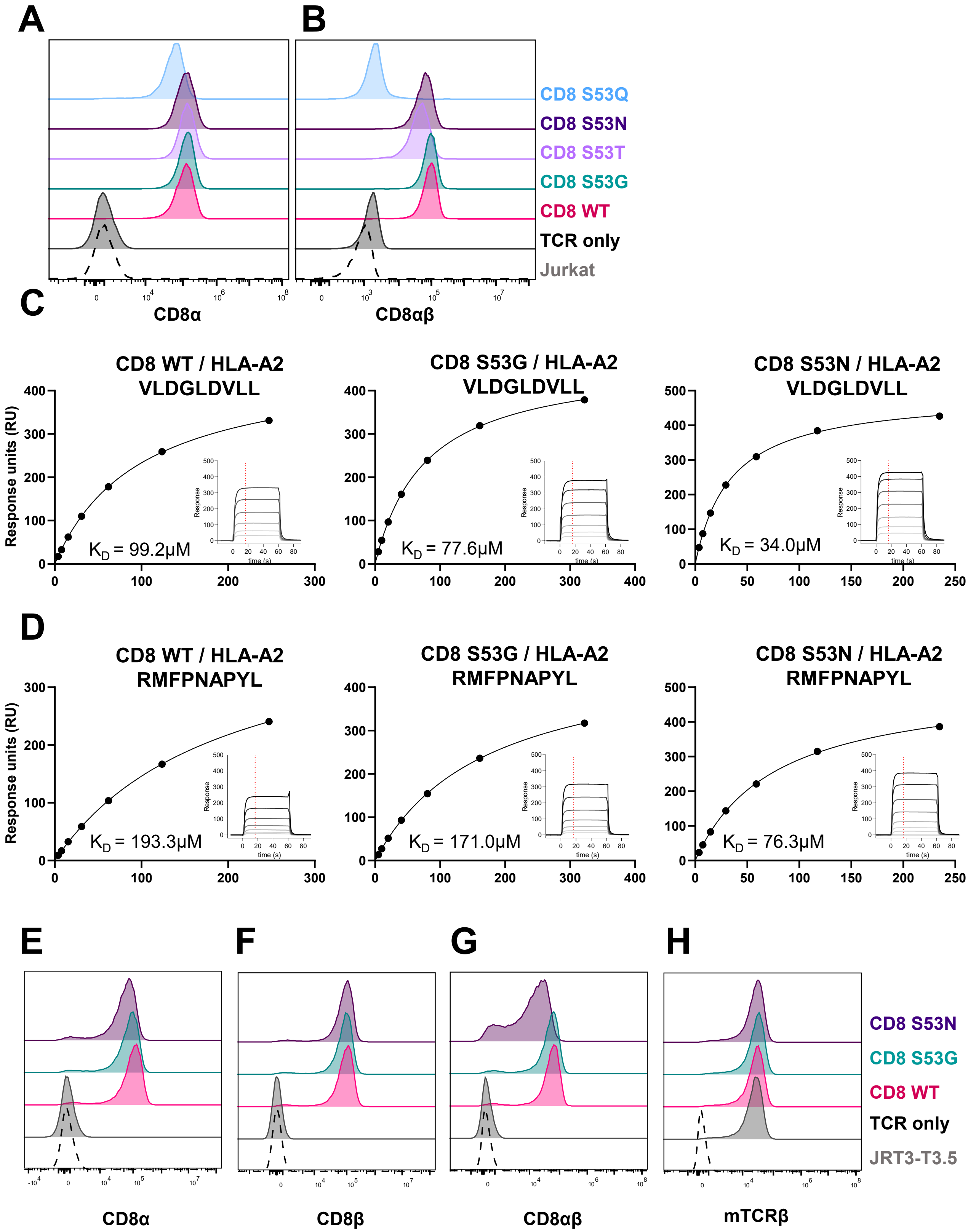

Supporting Figure S1 Biophysical and cellular characterization of CD8 variants.

A-B Expression of CD8α (A) and CD8αβ (B) on Jurkat cells transduced with the RLA TCR and CD8αβ containing either wild-type (WT) CD8α or mutated forms (S53G, S53T, S53N, or S53Q) of CD8α. **C- D** Representative surface plasmon resonance affinity measurements of wild-type (WT) CD8αα and the most functionally potent variants of CD8αα, namely S53G and S53N, versus VLD/HLA-A*0201 or RMF/HLA-A*0201. **E–H** Expression of CD8α (E), CD8β (F), CD8αβ (G), and TCRβ (H) on MEL5 TCR^{+} $CD8\alpha\beta^{+}$ JRT3-T3.5 cells transduced with CD8αβ containing either wild-type (WT) CD8α (red) or mutated forms of CD8α, namely S53G (teal) or S53N (purple).

S2 RLA TCR sequencing and affinity measurement

A

RLA TCR Sequence

TRAV	CDR3	TRAJ
19	CALSEAVTDSSYKLI	12
**TRBV**	**CDR3**	**TRBJ**
10-3	CATGQGEYNEQF	2-1

B

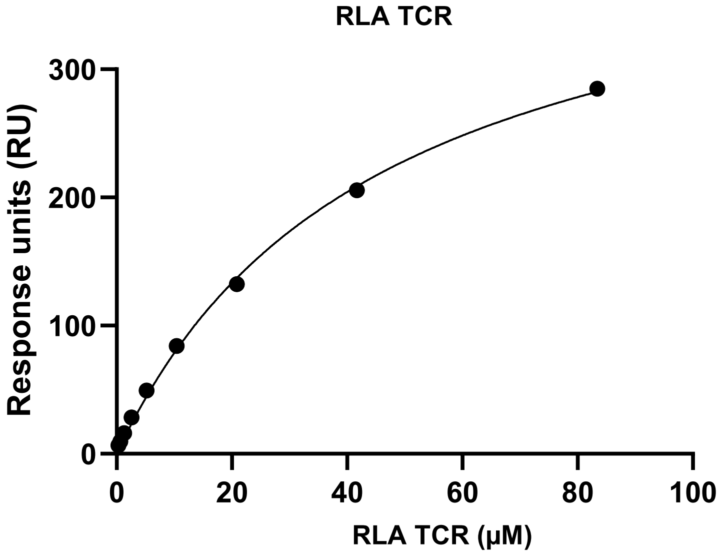

Supporting Figure S2. Sequencing and biophysical characterization of the RLA TCR

A TCRα and TCRβ sequences of the RLA TCR. Gene use was assigned according to the ImMunoGeneTics (IMGT) information system (http://www.imgt.org). **B** Surface plasmon resonance measurements of the RLA TCR versus RLA/HLA-A*0201.

S4 Sorting strategy for primary T-cells and further functional data in primary CD8 and CD4 T-cells

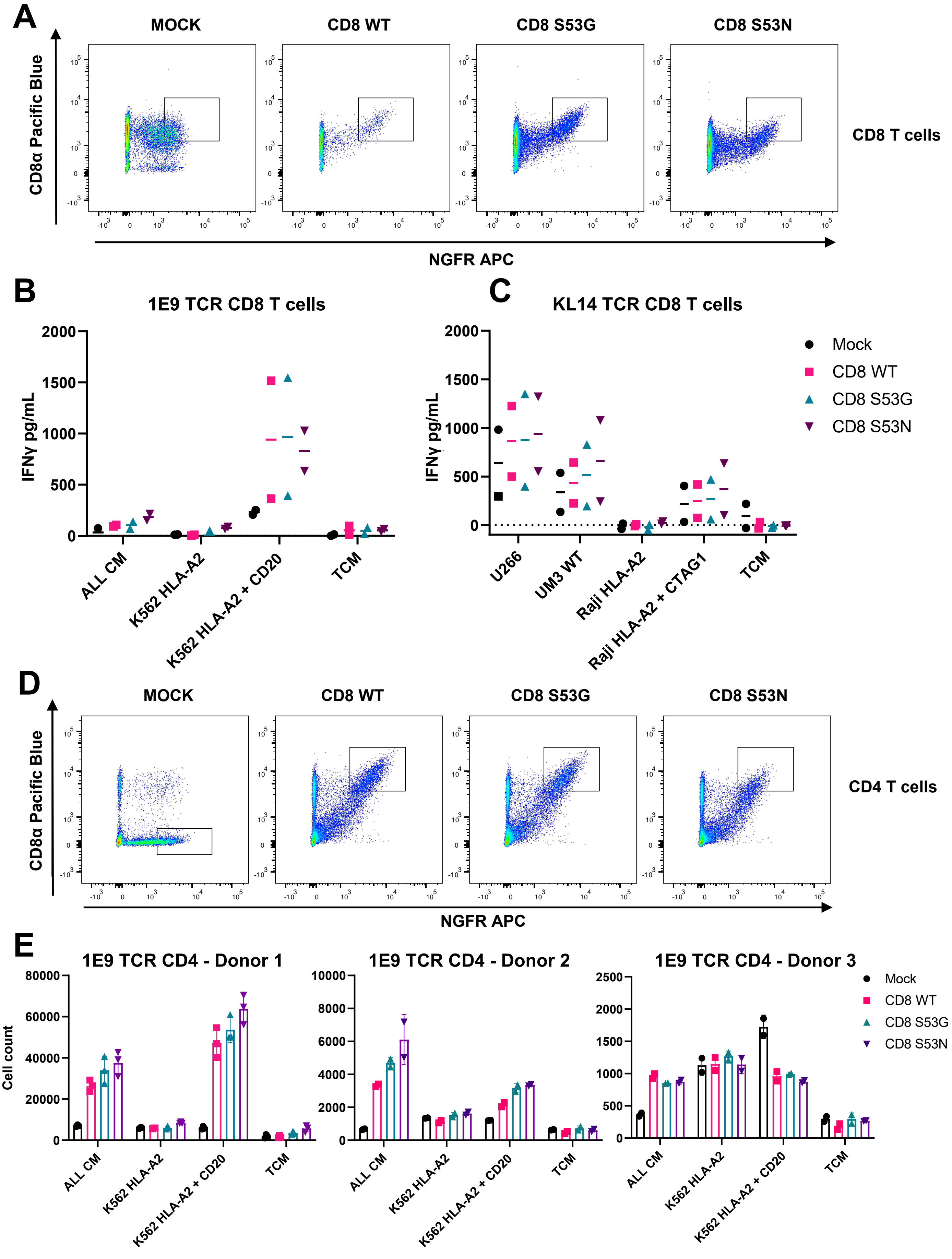

Supporting Figure S3 Functional analysis of primary CD4$^+$ and CD8$^+$ T cells transduced with tumor-targeting TCRs.

A Gating strategy for the purification of primary CD8$^+$ T cells expressing CD8αβ containing either wild-type (WT) CD8α or mutated forms of CD8α, namely S53G or S53N, alongside the 1E9 TCR via FACS. **B** Primary CD8$^+$ T cells expressing CD8αβ containing either wild-type (WT) CD8α (red) or mutated forms of CD8α, namely S53G (teal) or S53N (purple), alongside the 1E9 TCR were cocultured with a panel of cell lines lacking or expressing CD20. The panel shows mean IFN-γ production from each of two donors. **C** Gating strategy for the purification of primary CD4$^+$ T cells expressing CD8αβ containing either wild-type (WT) CD8α or mutated forms of CD8α, namely S53G or S53N, alongside the 1E9 TCR via FACS. **D** Primary CD8$^+$ T cells expressing CD8αβ containing either wild-type (WT) CD8α (red) or mutated forms of CD8α, namely S53G (teal) or S53N (purple), alongside the KL14 TCR were cocultured with a panel of cell lines lacking or expressing CTAG1. The panel shows mean IFN-γ production from each of two donors. **E** Primary CD4$^+$ T cells expressing CD8αβ containing either wild-type (WT) CD8α (red) or mutated forms of CD8α, namely S53G (teal) or S53N (purple), alongside the 1E9 TCR were cocultured with a panel of cell lines lacking or expressing CD20. Cells were quantified after 5 days via flow cytometry (n = 3 donors). TCM, T cell medium. Data are shown as mean ± SD from duplicate or triplicate measurements per donor.
